# Supplementary material for: An Italian Survey on Dietary Habits and Changes during the COVID-19 Lockdown
Source: Nutrients. 2021 Apr 5;13(4):1197. doi: 10.3390/nu13041197 (PMC8065756; doi:10.3390/nu13041197)
Supplement: Supplementary file 1 [file nutrients-13-01197-s001.pdf]

Supplementary materials:

Figure S1. Dietary habits and lifestyle changes during the COVID-19 pandemic period

20. How many portions of cereals do you consume per day?

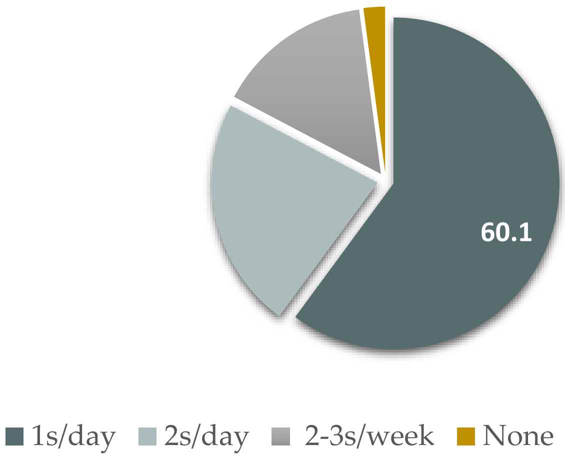

21. How many portions of cheese or dairy products do you consume per day?

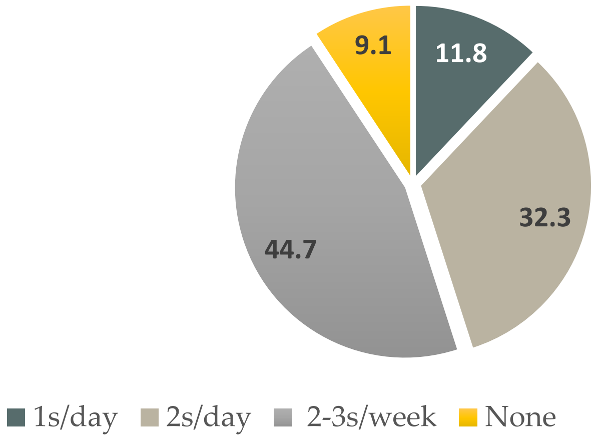

22. How many eggs do you consume per week?

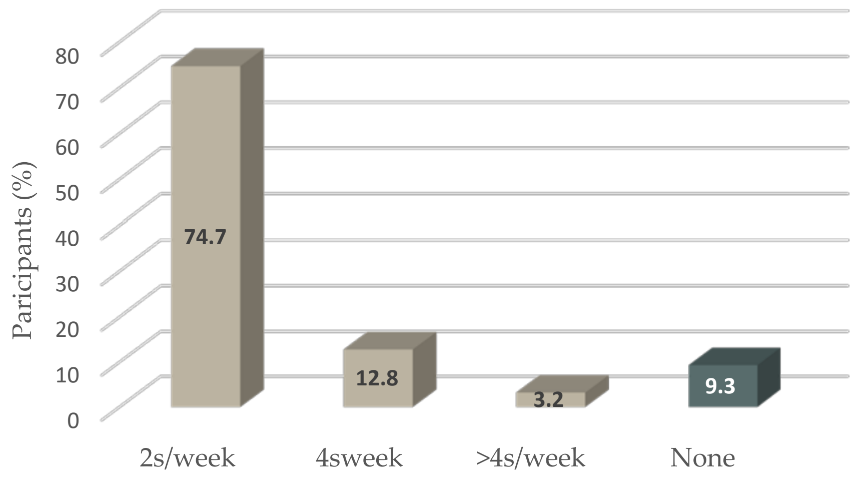

23. How many meals do you eat per day?

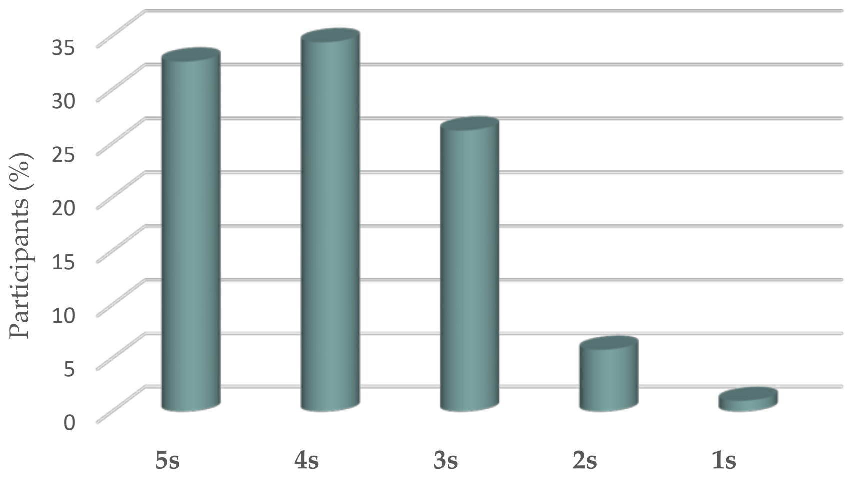

24. Have you eaten breakfast regularly?

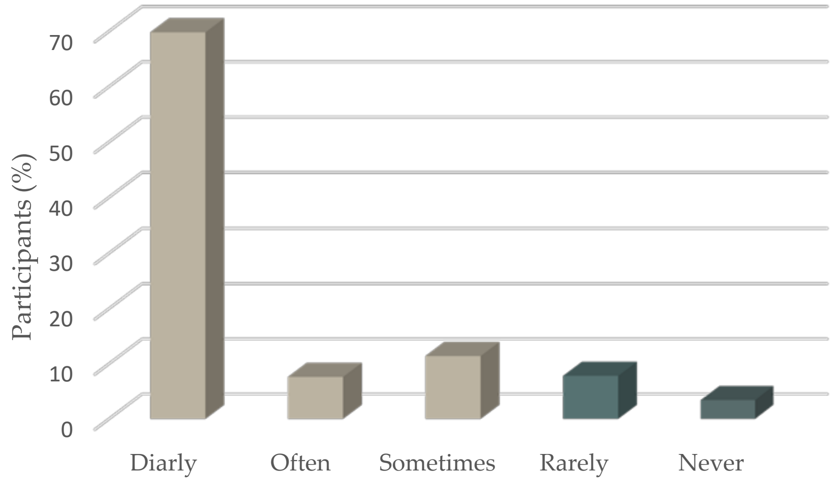

25. Which snack did you eat most often?

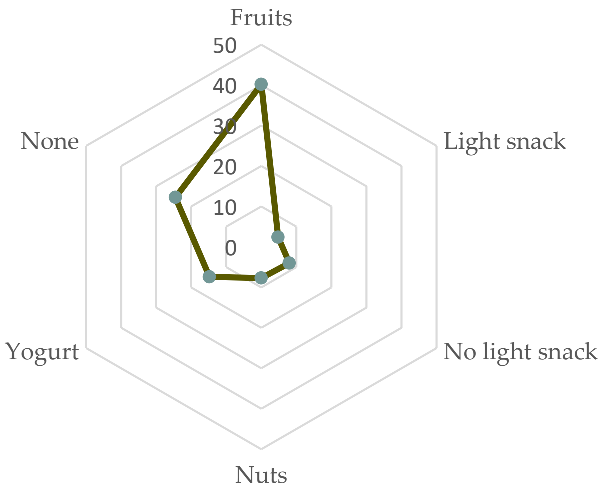

26. Have you consumed wholegrain products?

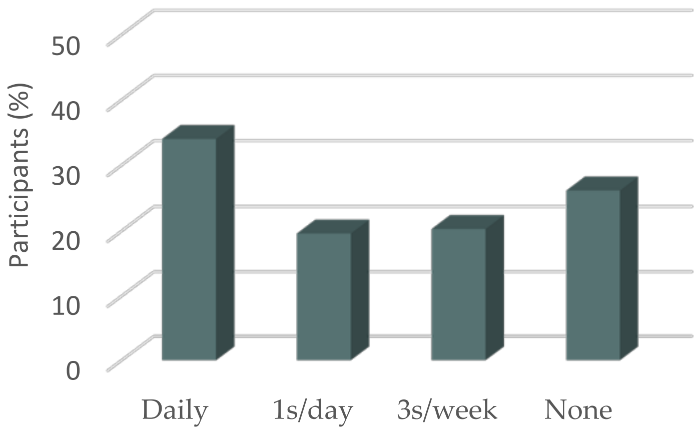

27. Has your consumption of frozen food increased?

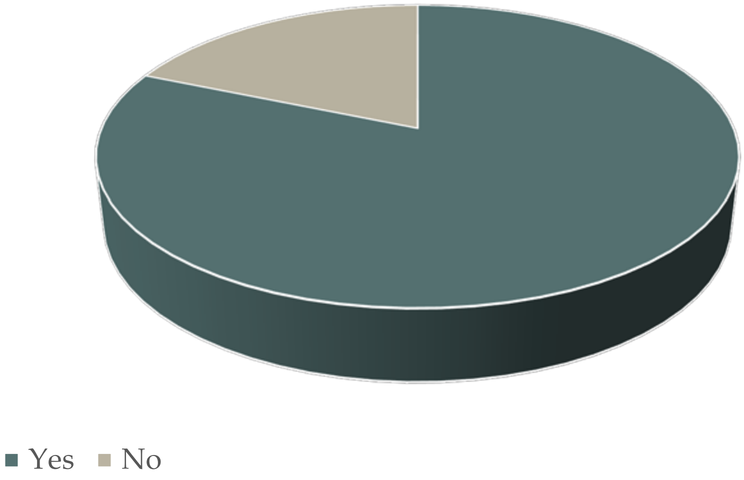

28. What was your main method of cooking food?

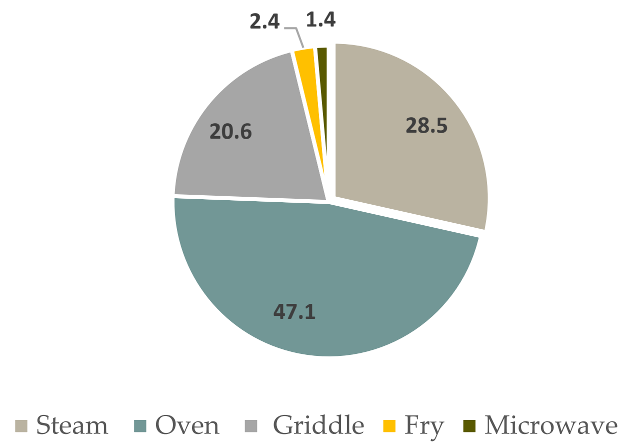

29. When do you often use frying mode?

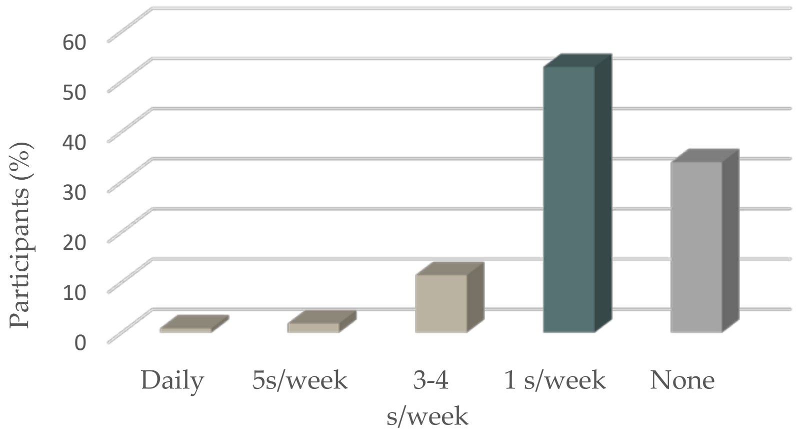

30. What was the water consumption during the COVID-19 pandemic period?

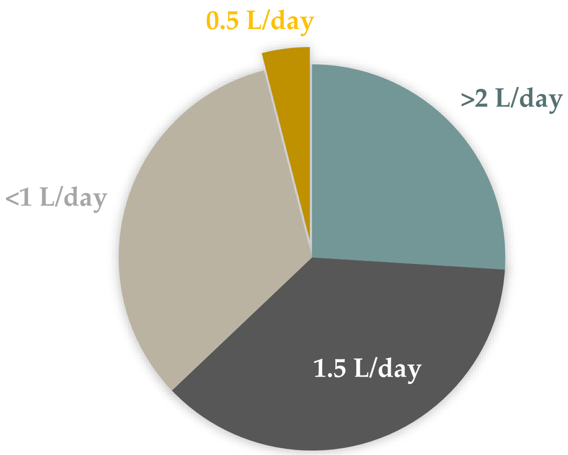

31. What type of water did you consume most frequently?

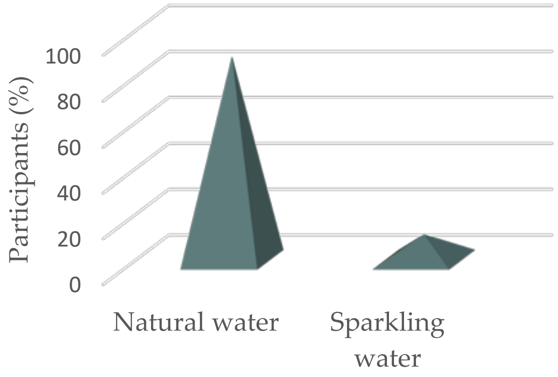

32. Have you consumed more fruit juices than before COVID-19 pandemic period?

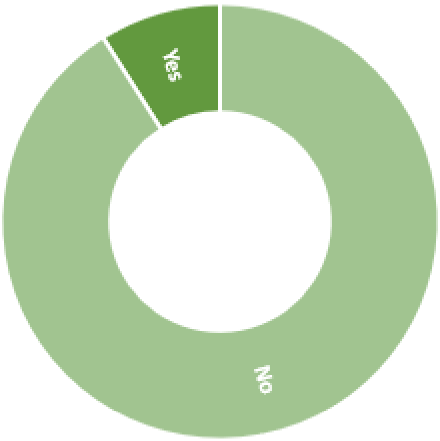

33. Has your alcohol consumption increased?

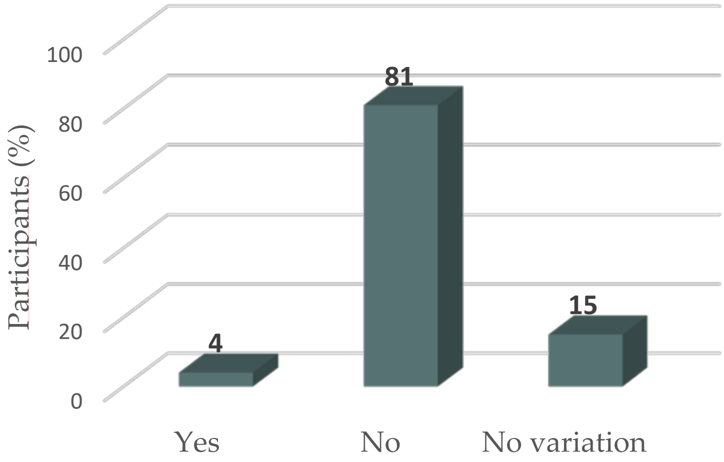

34. What was the alcohol consumption during the COVID-19 pandemic period?

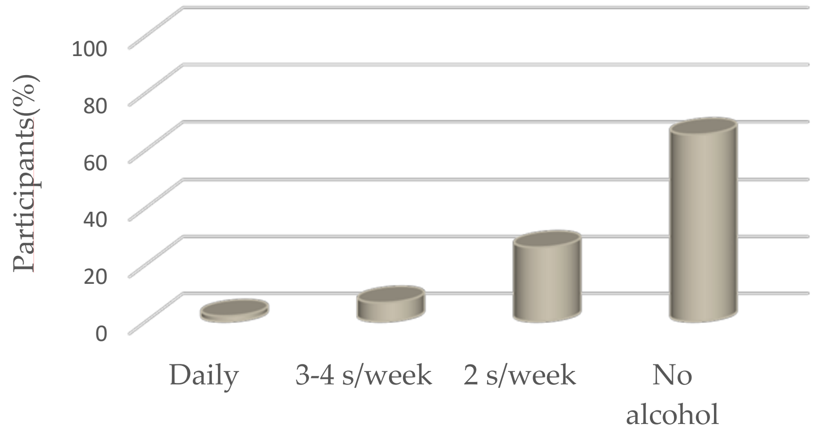

35. During this period, is your coffee consumption increased?

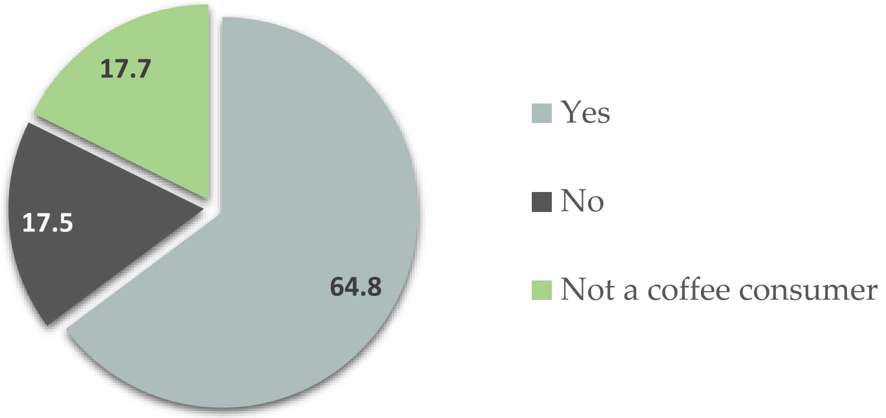

36. During this period, is sweet food comsumption increased?

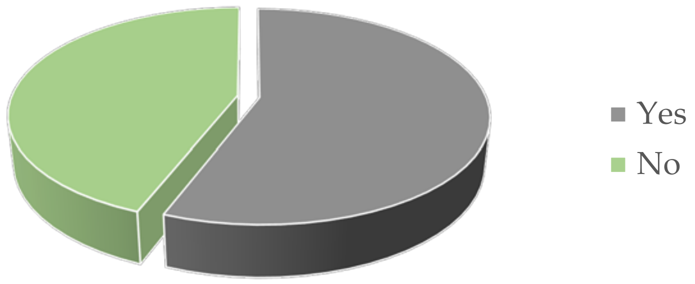

37. How do you consider yourself ?

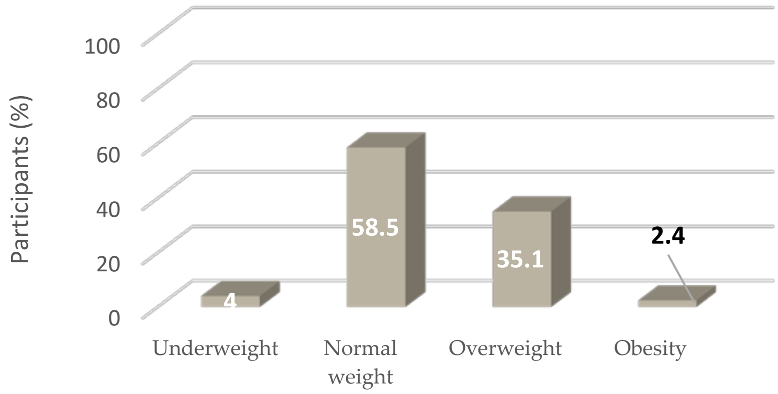

38. Did your eating habits changed during the COVID-19 pandemic period?

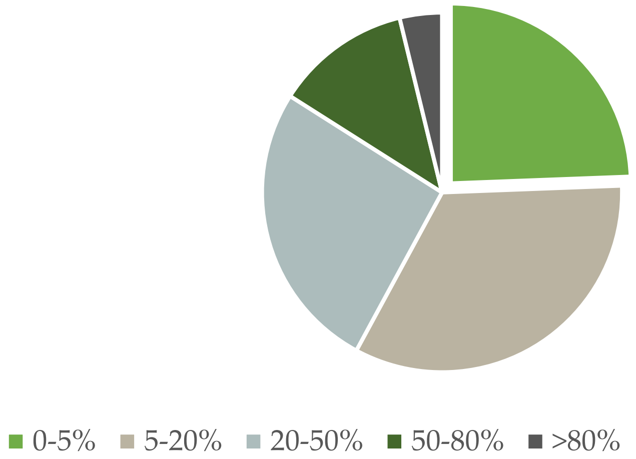

39. How much has your "food rude" increased?

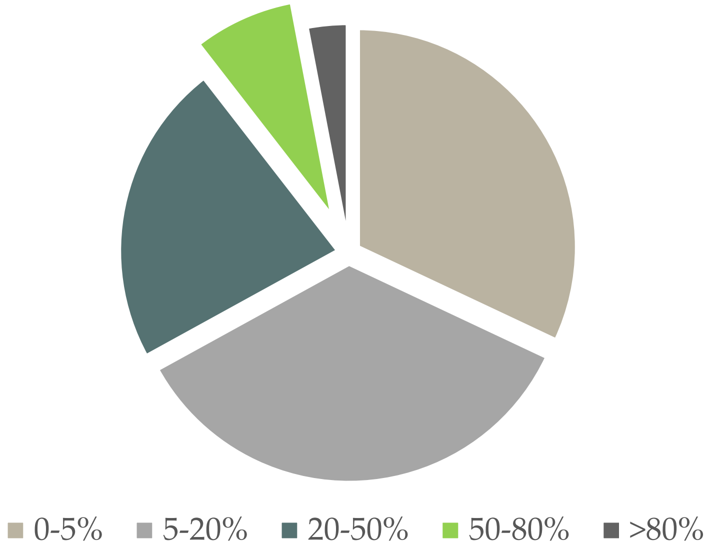

40. Did you change your weight during the COVID-19?

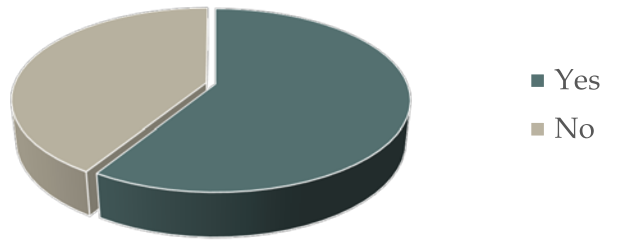

41. How has your weight changed?

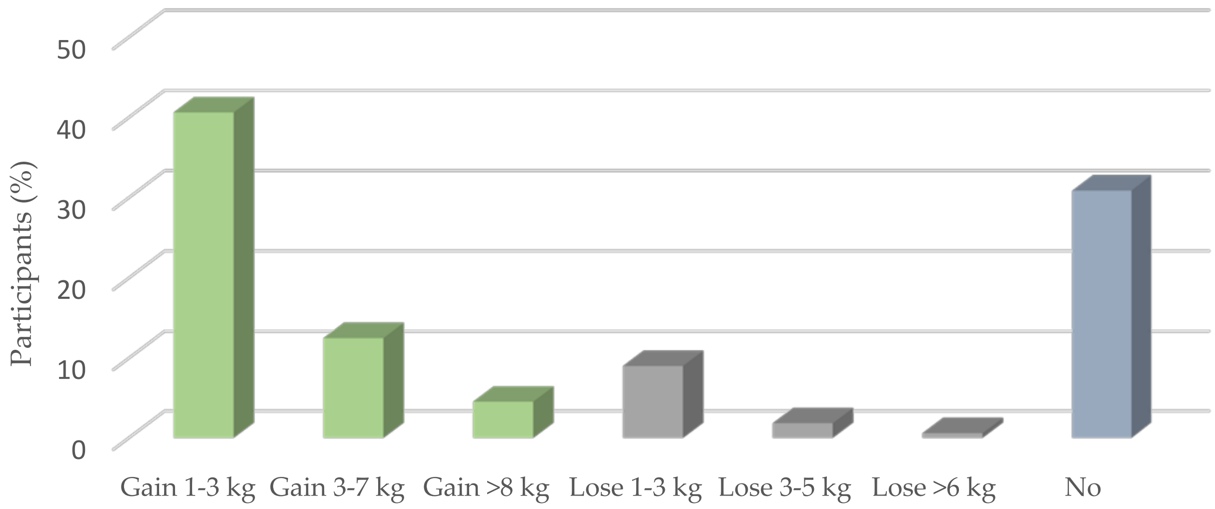

42. Did the frequency of your physical activity change during the pandemic period?

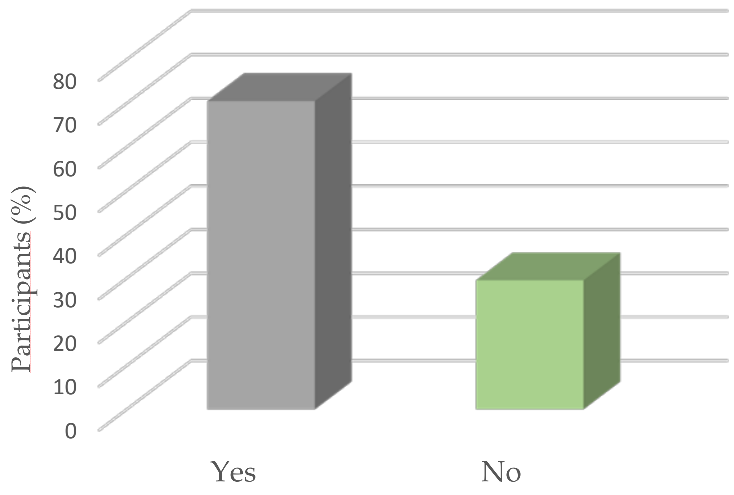

43. HOW MANY TIMES DO YOU PLAY SPORT AT HOME?

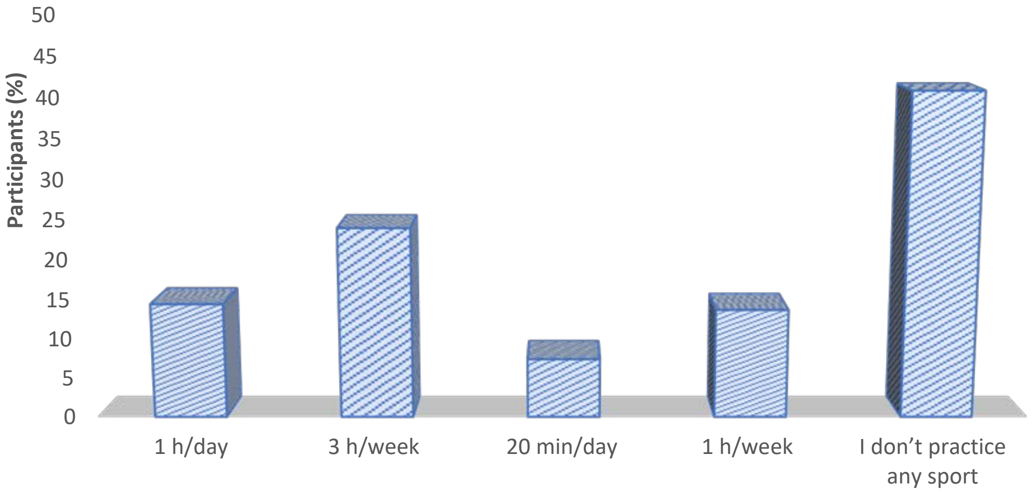

Figure S2. Scatter plots created with JASP (www.jasp-stats.org). (A) x-axis age, y-axis meals' number; (B) x-axis age, y-axis snack; (C) x-axis age, y-axis coffee consumption; (D) x-axis age, y-axis fruit juice consumption; (E) x-axis age, y-axis cereal consumption; (F) x-axis age, y-axis wholewheat products consumption; (G) x-axis age, y-axis sweets consumption; (H) x-axis age, y-axis eggs consumption. Gender is the split variable: female participants (1●), and male participants (2●).

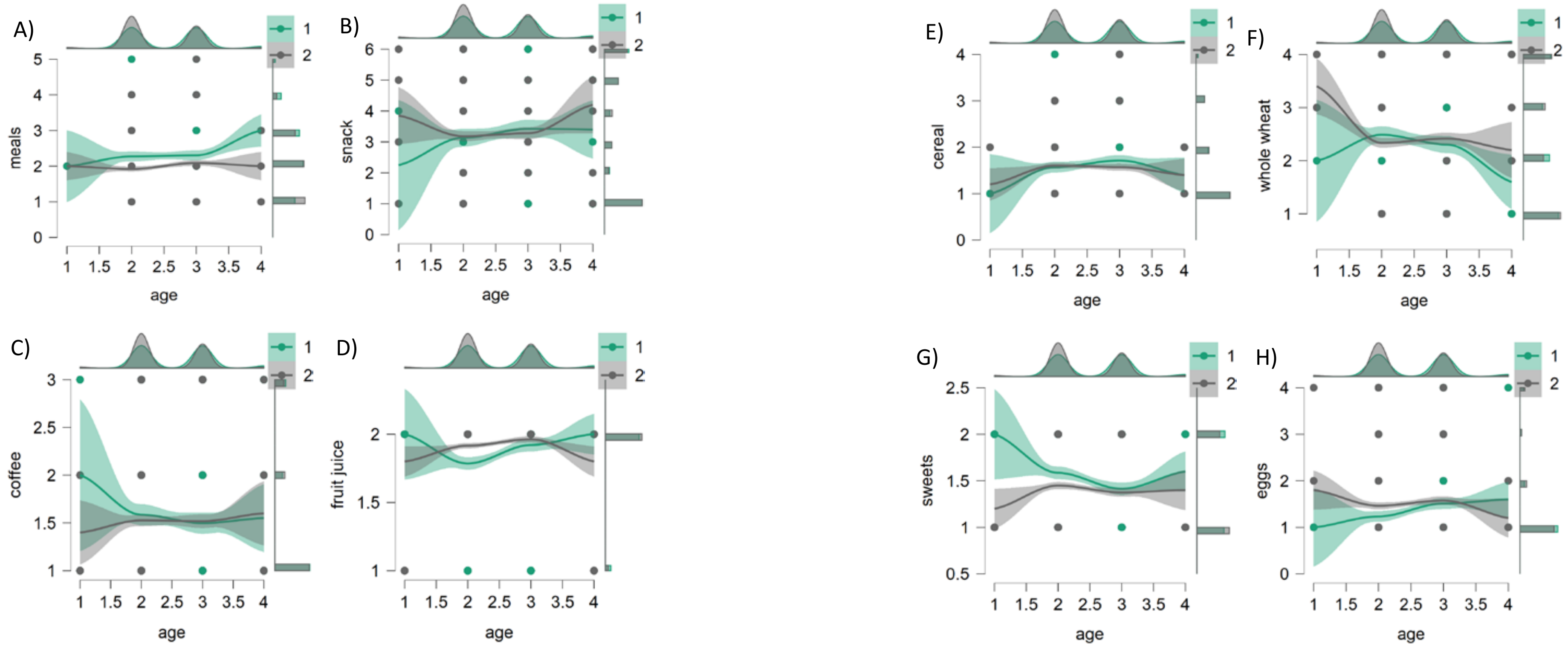

Meals' number 1 = 5s, 2 = 4s, 3 = 3s, 4 = 2s, 5 = 1s; ***Snack type*** 1 = fruits, 2 = light snack, 3 = no light snack, 4) nuts, 5 = yogurt, 6) none; ***Coffee consumption increase*** 1 = Yes; 2 = No; 3 = No variation; ***Fruit juice consumption*** 1 = Yes, 2 = No; ***Cereal portions*** 1 = 1s/day, 2 = 2s/day, 3 = 2-3s/week, 4 = None; ***Wholegrain consumption*** 1 = daily, 2 = 1s/day, 3 = 3s/week, 4 = None; ***Sweets consumption increase*** 1 = Yes, 2 = No; ***Eggs consumption*** 1 = 2s/week, 2 = 4sweek, 3 = >4s/week, 4 = None.
